# Seabuckthorn Leaves Extract and Flavonoid Glycosides Extract from Seabuckthorn Leaves Ameliorates Adiposity, Hepatic Steatosis, Insulin Resistance, and Inflammation in Diet-Induced Obesity

**DOI:** 10.3390/nu9060569

**Published:** 2017-06-02

**Authors:** Eun-Young Kwon, Jeonghyeon Lee, Ye Jin Kim, Ara Do, Ji-Young Choi, Su-Jung Cho, Un Ju Jung, Mi-Kyung Lee, Yong Bok Park, Myung-Sook Choi

**Affiliations:** 1 Department of Food Science and Nutrition, Kyungpook National University, 1370 San-Kyuk Dong Puk-Ku, Daegu 41566, Korea; savage20@naver.com (E.-Y.K.); wjdgus4411@naver.com (J.L.); freewilly59@hanmail.net (Y.J.K.); holy30000@hanmail.net (A.D.); jyjy31@hanmail.net (J.-Y.C.); chocrystalhihi@hanmail.net (S.-J.C.); 2 Center for Food and Nutritional Genomics Research, Kyungpook National University, 1370 San-Kyuk Dong Puk-Ku, Daegu 41566, Korea; 3 Department of Food Science and Nutrition, Pukyong National University, Busan 608-737, Korea; jungunju@naver.com; 4 Department of Food and Nutrition, Sunchon National University, Suncheon 540-950, Korea; leemk@sunchon.ac.kr; 5 School of Life Sciences and Biotechnology, Kyungpook National University, 1370 San-Kyuk Dong Puk-Ku, Daegu 41566, Korea; parkyb@knu.ac.kr

**Keywords:** flavonoid glycosides, hepatic steatosis, inflammation, insulin resistance, obesity, seabuckthorn

## Abstract

The aim of the current study was to elucidate the effect of seabuckthorn leaves (SL) extract and flavonoid glycosides extract from seabuckthorn leaves (SLG) on diet-induced obesity and related metabolic disturbances, and additionally, to identify whether flavonoid glycosides and other components in SL can exert a possible interaction for the prevention of metabolic diseases by comparing the effect of SL and SLG. C57BL/6J mice were fed a normal diet (ND, AIN-93G purified diet), high-fat diet (HFD, 60 kcal% fat), HFD + 1.8% (*w*/*w)* SL (SL), and HFD + 0.04% (*w*/*w*) SLG (SLG) for 12 weeks. In high fat-fed mice, SL and SLG decreased the adiposity by suppressing lipogenesis in adipose tissue, while increasing the energy expenditure. SL and SLG also improved hepatic steatosis by suppressing hepatic lipogenesis and lipid absorption, whilst also enhancing hepatic fatty acid oxidation, which may be linked to the improvement in dyslipidemia. Moreover, SL and SLG improved insulin sensitivity by suppressing the levels of plasma GIP that were modulated by secreted resistin and pro-inflammatory cytokine, and hepatic glucogenic enzyme activities. SL, especially its flavonoid glycosides (SLG), can protect against the deleterious effects of diet-induced obesity (DIO) and its metabolic complications such as adiposity, dyslipidemia, inflammation, hepatic steatosis, and insulin resistance.

## 1. Introduction

The global prevalence of overweight and obesity has increased every decade in a number of countries and has been described as a global pandemic [1,2]. In 2014, according to world health organization (WHO), more than 1.9 billion adults were overweight and over 600 million of them were obese. Obesity is defined as excessive fat accumulation and is associated with various obesity-related metabolic syndromes such as adiposity, dyslipidemia, insulin resistance, and non-alcoholic fatty liver disease (NAFLD). Moreover, about 20–30% of severe obese patients have been diagnosed with NAFLD, so it has become an emerging issue for healthcare management [3]. NAFLD is a modern society health problem which ranges from the simple accumulation of triglycerides in the hepatocytes with no inflammation (hepatic steatosis), to steatosis along with liver inflammation (non-alcoholic steatohepatitis, NASH). Although the underlying mechanisms among adiposity, NAFLD, insulin resistance, and inflammation are not fully understood, the dysregulation of lipid metabolism in liver and adipose tissue is associated with adiposity and its complications [4].

Seabuckthorn (*Hippophae rhamnoides* L.) is a plant material and is in the family Elaeagnaceae. Seabuckthron is native to Europe and Asia, and the majority of the seabuckthorn plant’s habitat is in northern Europe, China, Mongolia, Russia, and Canada. It is a unique and valuable plant currently cultivated in various parts of the world, and grows best in deep, well drained, sandy loam soil with ample organic matter. All parts of the seabuckthorn plant are considered to be a rich source of bioactive substances like isoflavones and flavonoids, which have various beneficial effects on health, such as anti-atherogenic, anti-oxidant, anti-cancer, and anti-bacterial effects [5]. In particular, its leaf extracts are reported to have marked anti-bacterial, anti-tumor, anti-inflammatory, and anti-oxidative activities [6,7,8]. This leaf extract contains a high content of flavonoid glycosides, including isorhamnetin 3-glucoside and quercetin 3-glucoside, which are known to prevent adiposity and dyslipidemia [9,10]. With the benefits of having various habitat and bioactive effects, seabuckthorn plays a significant part in the nutraceutical market. However, the potential anti-obesity effects of seabuckthorn leaves (SL) extract still remain unclear, and no studies have determined the effect of flavonoid glycosides extract from seabuckthorn leaves (SLG) on the lipid metabolism of adipose tissue and the liver in response to a high fat diet (HFD). Thus, the present study was undertaken to evaluate the effect of SL ethanol extract and flavonoid glycosides extract from SL (SLG) on adiposity, hepatic steatosis, insulin resistance, and inflammation in diet-induced obese (DIO) mice, and to identify whether flavonoid glycosides and other components in SL can exert a possible interaction for the prevention of metabolic diseases by comparing the effect of SL and SLG.

## 2. Materials and Methods 

### 2.1. Preparation of Seabuckthorn Leaves (SL) Extract and Flavonoid Glycosides Extract from SL (SLG)

The dried seabuckthorn (*Hippophae rhamnoides* L.) leaves (1.15 kg) were extracted twice with 80% aqueous EtOH (10 L) under an ultrasonic cleaner (Power Sonic 420, Hwashintech, Incheon, Korea) for 2 h, filtered, and evaporated under reduced pressure. The concentrated EtOH extract (430.12 g) was obtained and isolated, and purified the flavonoids as follows. A portion (50 g) of the extract was solubilized in 20% aqueous EtOH and successively loaded into a Diaion HP-20 (Mitsubishi Chem. Co., Tokyo, Japan) column (5.5 × 50 cm). The column was eluted successively with 20%, 30%, and 50% aqueous EtOH, and each fraction was then evaporated to yield 20% EtOH fraction (Fr.) (32.3 g), 30% EtOH fr. (6.9 g), and 50% EtOH Fr. (3.0 g), respectively. All fractions were monitored by a UV-vis spectrophotometer and analytical HPLC to ascertain flavonoids. Among three fractions, the 30% EtOH Fr. was chromatographed on a silica gel (70–230 mesh, Merck, Damstadt, Germany) column (10.5 × 70 cm) with CHCl_3_-MeOH-H_2_O (65:35:7, *v*/*v*) as an eluent and obtained four fractions; Fr. 1 (0.35 g), Fr. 2 (0.92 g), Fr. 3 (0.85 g), and Fr. 4 (0.51 g). Fr. 2 and Fr. 3 were successively chromatographed on a ODS-A (YMC Inc., MA, USA) column (4.5 × 60 cm) with 25% aqueous EtOH, and a Sephadex LH-20 column (2.5 × 80 cm) with 80% aqueous EtOH, and yielded isorhamnetin 3-glucoside (4.7 mg) from Fr. 2 and quercetin 3-glucoside (5.3 mg) from Fr. 3, respectively. Finally, the two flavonoids were identified by NMR analysis (Table 1), and a comparison of the spectral data and the literature values was conducted [11].

### 2.2. Experimental Animals and Diets

Male C57BL/6J mice (four-week-old) were obtained from The Jackson Laboratory (Bar Harbor, ME, USA). All mice were individually housed under a constant temperature (24 °C) and 12-h light/dark cycle, fed a normal chow diet for a one-week acclimation period, and subsequently randomly divided into four groups. The mice were fed a normal diet (ND, AIN-93G purified diet, *n* = 10), HFD (60% of kilocalories from fat, *n* = 10), HFD with 1.8% (*w*/*w*) of SL (*n* = 10), and HFD with 0.04% (*w*/*w*) of SLG (*n* = 10) for 12 weeks, respectively. A total of 1.8% (*w*/*w*) of SL contains 0.04% (*w*/*w*) of SLG. The experimental diets were prepared every week and stored at 4 °C. At the end of the experimental period, all mice were anesthetized with isoflurane (5 mg/kg body weight, Baxter, MN, USA) after 12 h of fasting. Blood was taken from the inferior vena cava to determine the plasma lipid, adipokine, and hormone concentrations. The liver and adipose tissue were removed, rinsed with physiological saline, weighed, immediately frozen in liquid nitrogen, and stored at −70 °C until analysis. The animal study protocols were approved by the Ethics Committee at Kyungpook National University (Approval No. KNU 2015-0020).

The energy expenditure, morphology of the liver and fat tissues, glucose metabolism markers, plasma lipid contents, hepatic and fecal lipid contents, glucose- and lipid-regulating enzyme activity, and analysis of gene expression were performed as stated in the Supplementary Materials on the materials and methods.

### 2.3. Energy Expenditure

Energy expenditure was measured using an indirect calorimeter (Oxylet; Panlab, Cornella, Spain). The mice were placed into individual metabolic chambers at 25 °C, with free access to food and water. O_2_ and CO_2_ analyzers were calibrated with highly purified gas standards. The oxygen consumption (*V*o_2_) and carbon dioxide production (*V*co_2_) were recorded at 3-min intervals using a computer-assisted data acquisition program (Chart 5.2; AD Instrument, Sydney, Australia) over a 24-h period, and the data were averaged for each mouse. Energy expenditure (EE) was calculated according to the following formula: EE (kcal/day/kg of body weight^0.75^) = *V*o_2_ × 1.44 × (3.815 + (1.232 × *V*o_2_/*V*co_2_)).

### 2.4. Morphology of the Liver and Fat Tissues

The liver and epididymal while adipose tissue (eWAT) were removed from each mouse. Samples were subsequently fixed in 10% (*v*/*v*) paraformaldehyde/phosphate-buffered saline and embedded in paraffin for staining with hematoxylin and eosin. Stained areas were visualized using a microscope set at 200× magnification.

### 2.5. Plasma Biomarkers

Plasma lipid concentrations were determined with commercially available kits. Plasma free fatty acid (FFA) levels were measured using the Wako enzymatic kit (Wako Chemicals, Richmond, VA, USA), and triglyceride, total cholesterol, HDL-cholesterol, glutamic oxaloacetic transaminase (GOT), and glutamic pyruvic transaminase (GPT) levels were determined using Asan enzymatic kits (Asan, Seoul, Korea). Plasma apolipoprotein AI (apo AI; Eiken, Japan) and apolipoprotein B (apo B; Eiken, Japan) levels were also measured using enzymatic kits. The values of nonHDL-cholesterol, the ratio of HDL-cholesterol to total cholesterol (HTR), and the atherogenic index (AI) were calculated as follow: nonHDL-cholesterol = ((total-cholesterol) − (HDL-cholesterol) − (triglyceride/5)), HTR (%) = (HDL-cholesterol/total-cholesterol) × 100, AI = ((total-cholesterol) – (HDL-cholesterol))/(HDL-cholesterol). Plasma insulin, incretin hormone gastric inhibitory polypeptide (GIP), adipokines (resistin, leptin, and adiponectin), cytokines (tumor necrosis factor alpha (TNF-α), interleukin 1β (IL-1β), IL-6, and plasminogen activator inhibitor-1 (PAI-1)) were determined with a multiplex detection kit from Bio-Rad (Hercules, CA, USA). All samples were assayed in duplicate and analyzed with a Luminex 200 Labmap system (Luminex, Austin, TX, USA). Data analyses were done with the Bio-Plex Manager software version 4.1.1 (Bio-Rad, Richmond, CA, USA).

### 2.6. Fasting Blood Glucose, Intraperitoneal Glucose Tolerance Test, and Homeostatic Index of Insulin Resistance

The blood glucose concentration was measured by the glucose oxidase method using a glucose analyzer (Glucocard, Arkray, Japan) in whole blood obtained from the tail vein after food withholding for 12 h. The intraperitoneal glucose tolerance test (IPGTT) was performed at week 11. After 12 h of fasting, the mice were injected intraperitoneally with glucose (0.5 g/kg of body weight). The blood glucose level was determined from the tail vein at 0, 30, 60, and 120 min after the glucose injection. The homeostatic index of insulin resistance (HOMA-IR) was calculated according to the homeostasis assessment model as follows: HOMA-IR = (fasting glucose (mmol/L) × fasting insulin (IU/mL))/22.51.

### 2.7. Hepatic and Fecal Lipid Contents

Hepatic and fecal lipids were extracted as previously described [12], and then dried lipid residues were dissolved in 1 mL of ethanol for the triglyceride, cholesterol, and fatty acid (FA) assays. Triton X-100 and a sodium cholate solution in distilled water were added to 200 μL of a dissolved lipid solution for emulsification. Hepatic and fecal triglyceride, cholesterol, and FA contents were analyzed with the same enzymatic kits that were used for the plasma analysis.

### 2.8. Preparation of Hepatic Subcellular Fractions

Hepatic and adipocyte mitochondrial, cytosolic, and microsomal fractions were prepared as previously described [13]. The mitochondrial fraction was used to measure glucose-6-phosphatase (G6Pase) and β-oxidation, and the cytosolic fraction was used to measure glucose-6-phosphate dehydrogenase (G6PD), malic enzyme (ME), fatty acid synthase (FAS), glucokinase, and phosphoenolpyruvate carboxykinase (PEPCK) activities. The microsomal fraction was used to measure phosphatidate phosphohydrolase (PAP) and acyl-CoA:cholesterolacyltransferase (ACAT) activities. The protein concentrations were determined using the Bradford method.

### 2.9. Glucose- and Lipid-Regulating Enzyme Activity

Glucose-6-phosphate dehydrogenase (G6PD) [14], fatty acid synthase (FAS) [15], malic enzyme (ME) [16], and phosphatidate phosphohydrolase (PAP) [17] activities were measured as previously described. Glucose-6-phosphatase (G6Pase) activity was determined using the method of Alegre et al. [18]. Phosphoenolpyruvate carboxykinase (PEPCK) activity was monitored in the direction of oxaloacetate synthesis using a spectrophotometric assay developed by Bentle and Lardy [19]. Fatty acid β-oxidation was measured spectrophotometrically by monitoring the reduction of NAD to NADH in the presence of palmitoyl-CoA as described by Lazarow [15], with a slight modification.

### 2.10. Analysis of Gene Expression

The liver tissues were homogenized in the TRIzol reagent (Invitrogen, Grand Island, NY, USA), and the total RNA was isolated according to the manufacturer’s instructions. The total RNA was converted to cDNA using the QuantiTect Reverse Transcription kit (Qiagen Gmbh, Hilden, Germany). mRNA expression was quantified by a quantitative real-time polymerase chain reaction (PCR) using the QuantiTect SYBR Green PCR kit (Qiagen) and SDS7000 sequence detection system (Applied Biosystems, CA, USA). Each cDNA sample was amplified using primers for the glyceraldehyde-3-phosphate dehydrogenase (GAPDH) gene labeled with SYBR green dye. 

The amplification was performed as follows: 10 min at 90 °C, 15 s at 95 °C, and 60 s at 60 °C for a total of 40 cycles. The cycle threshold (Ct) was defined as the cycle at which a statistically significant increase in the SYBR green emission intensity occurred. The Ct data were normalized relative to those for the housekeeping gene, GAPDH, which is stably expressed in mice. The relative gene expression was calculated with the 2^∆∆Ct^ method [20].

### 2.11. Primer

The primer were designed using a Primer 5.0 software (Primer-E Ltd., Plymouth, UK), SREBP1c (Forward: 5′-GGA GCC ATG GAT TGC ACA TT-3′, Reverse: 5′-CCT GTC TCA CCC CCA GCA TA-3′), CPT1α (Forward: 5′-ATC TGG ATG GCT ATG GTC AAG GTC-3′, Reverse: 5′-GTG CTG TCA TGC GTT GGA AGT C-3′), ABCG5 (Forward: 5′-TCA ATG AGT TTT ACG GCC TGA A-3′, Reverse: 5′-GCA CAT CGG GTG ATT TAG CA-3′), ABCG8 (Forward: 5′-GCA ATG CCC TCT ACA ACT CCT T-3′, Reverse: 5′-GAG GAA CGA CAG CTT GGA GAT C-3′), IRS2 (Forward: 5′-CCC ATG TCC CGC CGT GAA G-3′, Reverse: 5′-CTC CAG TGC CAA GGT CTG AAG G-3′), and GAPDH (Forward: 5′-ACA ATG AAT ACG GCT ACA GCA ACA G-3′, Reverse: 5′-GGT GGT CCA GGG TTT CTT ACT CC-3′).

### 2.12. Statistical Analysis

The parameter values were expressed as the mean (standard error of the mean (SEM)). Significant differences between the ND and HFD groups were determined by a student’s *t*-test and significant differences among the HFD, SL, and SLG groups were determined by one-way ANOVA using the SPSS program (SPSS Inc., Chicago, IL, USA). The results were considered statistically significant at *p* < 0.05.

## 3. Results and Discussion

### 3.1. SL and SLG Supplement Lowered Body Weight Gain and Improved Plasma Lipid Profiles in DIO Mice

**Figure 1:**
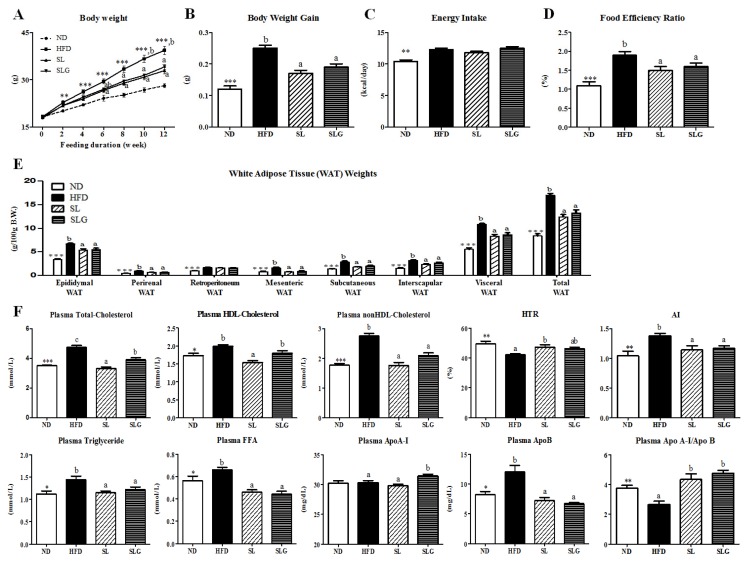
Effect of SL and SLG on body weight (**A**), body weight gain; (**B**), food intake; (**C**), food efficiency ratio; (**D**), white adipose tissue weights; (**E**) and plasma lipids levels; (**F**) in C57BL/6J mice fed HFD for 12 weeks. Data are shown as the mean ± SEM (*n* = 10). Significant differences between HFD versus ND are indicated; * *p* < 0.001, ** *p* < 0.01, *** *p* < 0.001. ^abc^ Means not sharing a common superscript are significantly different among the high-fat diet fed groups (HFD, SL, and SLG groups) at *p* < 0.05. ND, normal diet group; HFD, high-fat diet group; SL, HFD + 1.8% (*w*/*w*) ethanol extract of sea buckthorn leaves group; SLG, HFD + 0.04% (*w*/*w*) ethanol extract of flavonoid glycosides from sea buckthorn leaves group; Food Efficiency Ratio, body weight gain/Energy intakes per day; HTR, ratio of HDL-cholesterol to total cholesterol; AI, atherogenic index; FFA, free fatty acid; ApoA-I, apolipoprotein A-I; ApoB, apolipoprotein B.

HFD generally induces adiposity, hepatic steatosis, and insulin resistance through multiple mechanisms. We also observed that HFD (60.3% energy from fat) feeding for 12 weeks promoted the development of obesity, as indicated by significant increases in body weight (BW), BW gain, and body fat mass, with increased energy intake (Figure 1A–E). The supplementation of SL and SLG significantly decreased BW after six weeks and eight weeks of high-fat feeding, respectively, without altering the energy intake (Figure 1A–D). Both SL and SLG also resulted in a significant decrease in weights for all white adipose tissue (WAT) depots (epididymal, perirenal, mesenteric, subcutaneous, and interscapular WAT), except for retroperitoneum WAT, which led to a decrease in the visceral WAT and total WAT weights compared to the HFD group (Figure 1E). Thus, it is plausible that both SL and SLG suppressed BW gain by regulating the expansion of fat mass. We also found that SL and SLG supplementation improved dyslipidemia by decreasing the levels of plasma total-cholesterol, nonHDL-cholesterol, triglyceride, FFA, ApoB, and AI, while increasing the Apo A-I/Apo B ratio compared to the HFD group (Figure 1F). This finding is supported by a previous study [21], which demonstrated the body fat and plasma lipid level lowering effects of powdered SL via the regulation of lipid and antioxidant metabolism in DIO mice.

### 3.2. SL and SLG Supplement Lowered Adiposity by Decreasing Lipogenesis in Adipose Tissue, While Increasing Energy Expenditure in DIO Mice

**Figure 2:**
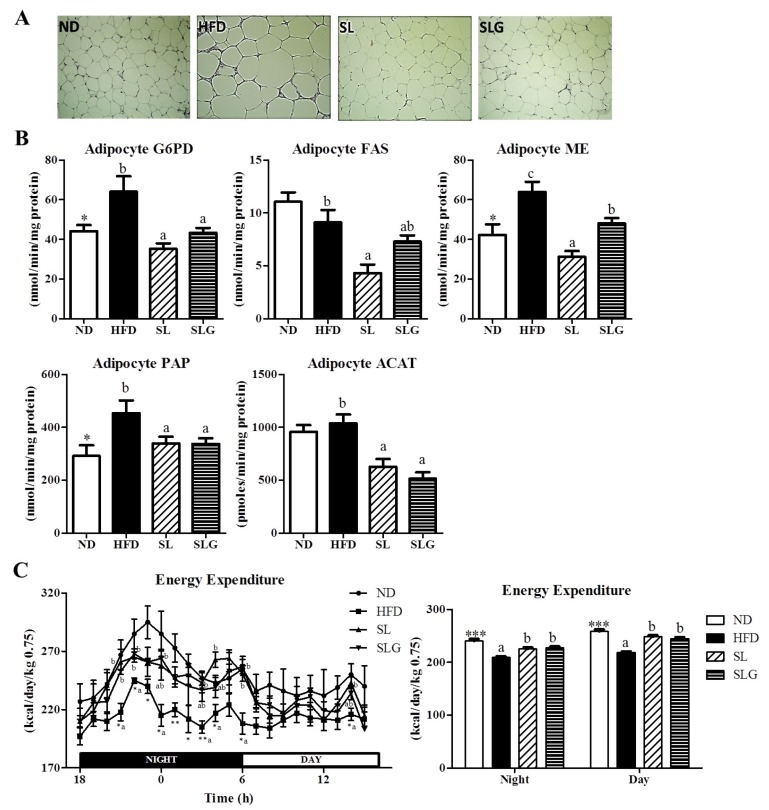
Effect of SL and SLG on adipocyte morphology (**A**) the activities of adipocyte lipogenic enzymes; (**B**) and energy expenditure in C57BL/6J mice fed HFD for 12 weeks. Data are shown as the mean ± SEM (*n* = 10). Significant differences between HFD versus ND are indicated; * *p* < 0.001, *** *p* < 0.001. ^abc^ Means not sharing a common superscript are significantly different among the high-fat diet fed groups (HFD, SL, and SLG groups) at *p* < 0.05. ND, normal diet group; HFD, high-fat diet group; SL, HFD + 1.8% (*w*/*w*) ethanol extract of sea buckthorn leaves group; SLG, HFD + 0.04% (*w*/*w*) ethanol extract of flavonoid glycosides from sea buckthorn leaves group; G6PD, glucose-6-phosphate dehydrogenase; FAS, fatty acid synthase; ME, malic enzyme; PAP, phosphatidate phosphohydrolase; ACAT, acyl-CoA:cholesterolacyltransferase.

Lee et al. [21] and Pichiah et al. [22] demonstrated that the supplementation of powdered SL or SL ethanol extract effectively suppressed BW gain and the expansion of adipose tissue mass by modulating the plasma leptin level and hepatic lipid metabolism. However, these previous studies have not analyzed the markers associated with lipid metabolism in adipose tissue, despite the reduced body fat mass induced by the SL supplement. Thus, we measured the activities of enzymes for lipogenesis in epididymal fat and found that G6PD, ME, PAP, and ACAT enzyme activities were suppressed by SL and SLG supplements compared to the HFD group, which is likely associated with the reduced adiposity (Figure 2A,B). Notably, SL supplementation also markedly diminished the activities of FAS compared to the HFD group. Interestingly, SL and SLG supplements led to an increase in the reduced energy expenditure by HFD during both the light phase and dark phase (Figure 2C). These observations indicate that SL and SLG have the potential to regulate adipocyte lipid metabolism and energy expenditure, thereby ameliorating adiposity in DIO mice.

### 3.3. SL and SLG Supplement Lowered the Levels of Hepatic Lipids and Lipotoxicity Markers by Modulating Hepatic Lipid Regulating Enzume Activities and Gene Expressions, and Increasing Fecal Lipids in DIO Mice

**Figure 3:**
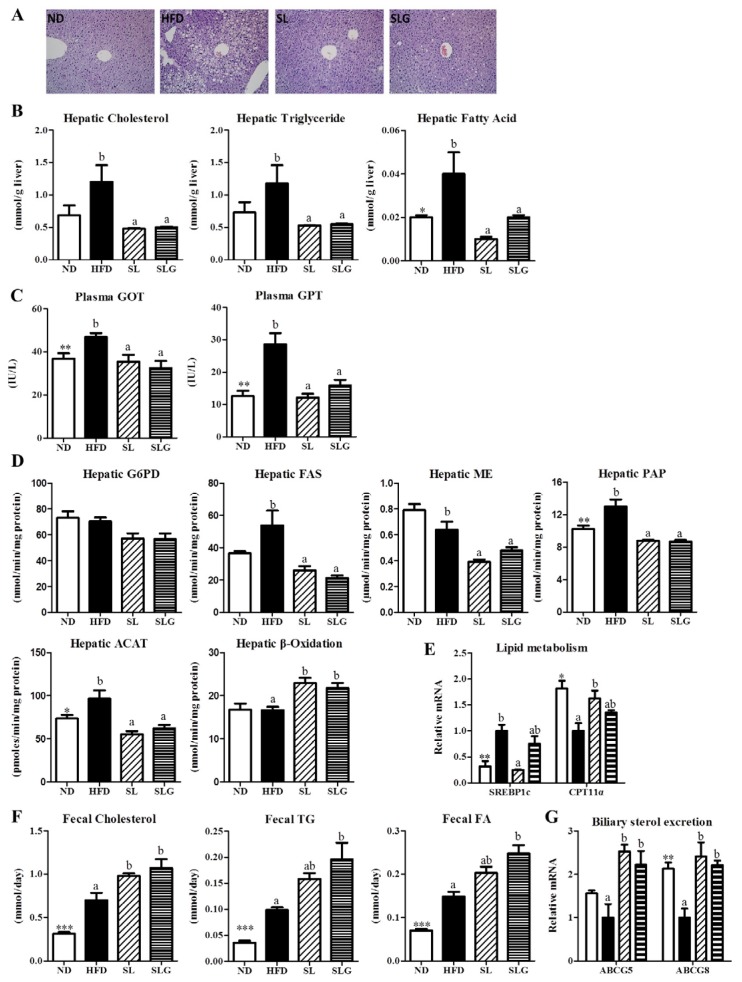
Effect of SL and SLG on hepatic morphology (**A**) hepatic lipids contents; (**B**) hepatic lipotoxicity markers; (**C**) hepatic lipid regulating enzyme activities; (**D**) and gene expressions; (**E**) fecal lipids contents; (**F**) and hepatic gene expression related with biliary sterol excretion in C57BL/6J mice fed HFD for 12 weeks. Data are shown as the mean ± SEM (*n* = 10). Significant differences between HFD versus ND are indicated; * *p* < 0.001, ** *p* < 0.01, *** *p* < 0.001. ^ab^ Means not sharing a common superscript are significantly different among the high-fat diet fed groups (HFD, SL, and SLG groups) at *p* < 0.05. ND, normal diet group; HFD, high-fat diet group; SL, HFD + 1.8% (*w*/*w*) ethanol extract of sea buckthorn leaves group; SLG, HFD + 0.04% (*w*/*w*) ethanol extract of flavonoid glycosides from sea buckthorn leaves group; GOT, glutamic oxaloacetic transaminase; GPT, glutamic pyruvic transaminase.

In general, a reduction of body fat mass and an improvement in dyslipidemia are highly correlated with improved hepatic steatosis [4,23]. SL and SLG supplementation improved hepatic steatosis, as well as adiposity, as evidenced by the reduced hepatic lipids accumulation and lipotoxicity markers (plasma GOT and GPT) compared with the HFD group (Figure 3A–C). SL and SLG supplementation markedly suppressed the hepatic lipogenic enzyme activities (FAS, ME, PAP, ACAT) and *SREBP1c* gene expression, and elevated the hepatic β-oxidation enzyme activity and *CPT1α* gene expression compared to the HFD group (Figure 3D,E), suggesting that SL and SLG may limit hepatic lipid availability by inhibiting lipogenesis and increasing fatty acid oxidation, thereby reducing hepatic lipotoxicity. Moreover, SLG supplementation significantly elevated fecal cholesterol, triglyceride, and FA levels with the mRNA expression of hepatic *ABCG5* and *ABCG8* (Figure 3F,G). Similarly, SL supplementation significantly increased fecal cholesterol and hepatic *ABCG5* and *ABCG8* mRNA expressions (Figure 3F,G). These could contribute to the inhibition of the hepatic lipid load by promoting biliary sterol excretion and decreasing the absorption of dietary fat.

### 3.4. SL and SLG Improved Insulin Resistance and Glucose Tolerance by Modulating Activities of Hepatic

#### Glucose-Regulating Enzymes and Levels of Plasma Adipokines and Cytokines in DIO Mice

**Figure 4:**
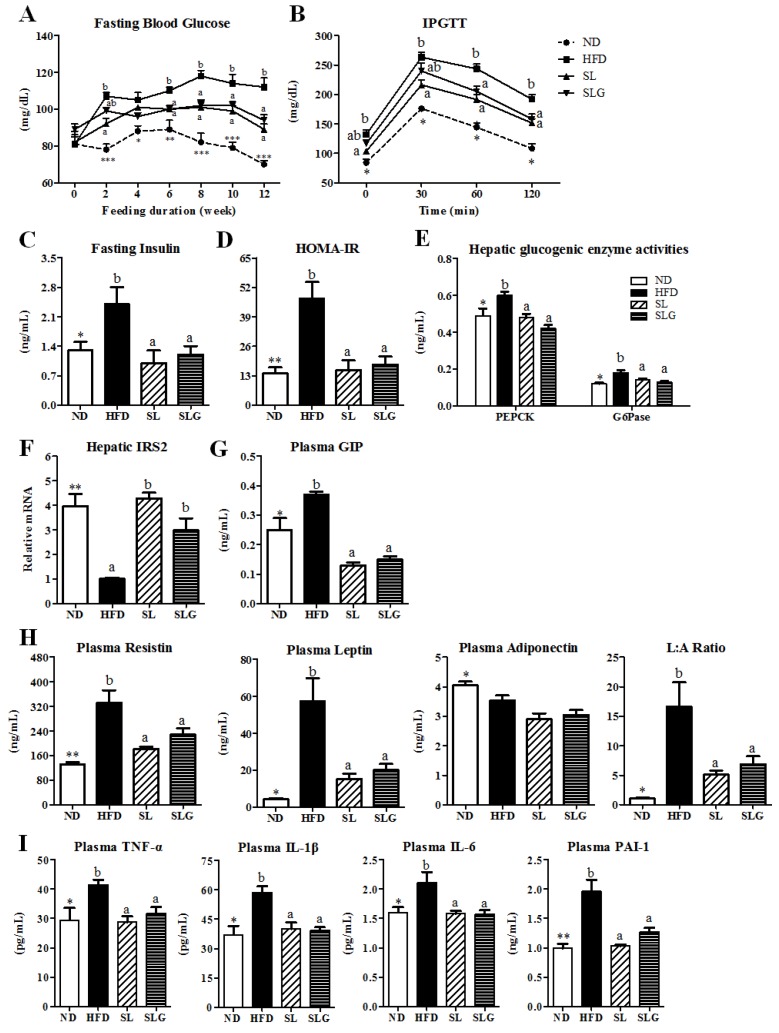
Effect of SL and SLG on fasting blood glucose (**A**) IPGTT; (**B**), fasting insulin; (**C**) HOMA-IR; (**D**) hepatic glucogenic enzymes; (**E**) hepatic IRS2 gene; (**F**) plasma GIP; (**G**) plasma adipokines; (**H**) and plasma pro-inflammatory cytokines in C57BL/6J mice fed HFD for 12 weeks. Data are shown as the mean ± SEM (*n* = 10). Significant differences between HFD versus ND are indicated; * *p* < 0.001, ** *p* < 0.01, *** *p* < 0.001. ^ab^ Means not sharing a common superscript are significantly different among the high-fat diet fed groups (HFD, SL, and SLG groups) at *p* < 0.05. ND, normal diet group; HFD, high-fat diet group; SL, HFD + 1.8% (*w*/*w*) ethanol extract of sea buckthorn leaves group; SLG, HFD + 0.04% (*w*/*w*) ethanol extract of flavonoid glycosides from sea buckthorn leaves group; IPGTT, intraperitoneal glucose tolerance test; HOMA-IR, homeostasis model assessment-estimated insulin resistance; PEPCK, phosphoenolpyruvate carboxykinase; G6Pase, glucokinase, glucose-6-phosphatase; IRS2, insulin receptor substrate 2; GIP, incretin hormone gastric inhibitory polypeptide; L:A Ratio, leptin:adiponectin ratio; TNF-α, tumor necrosis factor α; IL, interleukin; PAI-1, plasminogen activator inhibitor-1.

The striking improvement of hepatic steatosis coupled with the decreased adiposity in SL- and SLG- treated mice was associated with a normalization of the plasma glucose and insulin levels, which was a reflection of improved hepatic insulin sensitivity, as evidenced by the IPGTT and the reduced HOMA-IR (Figure 4A–D). In addition, SL and SLG supplementations suppressed the gluconeogenesis, as indicated by decreased hepatic G6Pase and PEPCK activities and the increased expression of hepatic *IRS2* mRNA (Figure 4E,F), which could be associated with the improved hepatic insulin sensitivity observed in SL- and SLG-supplemented DIO mice, similar to previous studies [17,18]. 

The incretin hormone GIP is a peptide hormone produced by the intestinal K cell and it acts directly on pancreatic islets to stimulate insulin secretion [24,25]. Fats strongly enhance GIP secretion [26], and its concentrations in obesity or obese type 2 diabetes mellitus (T2DM) patients are elevated [27]. GIP, in the presence of insulin, induces fatty acid uptake into adipose tissue and GIP receptor (GIPR)-deficient mice on HFD showed not only improved obesity by increasing the energy expenditure, but also insulin sensitivity, without differences in the energy intake compared to that of control mice [28]. Additionally, recent studies demonstrated that the binding of GIP to GIPR in the 3T3-L1 cells and adipose tissue of rats results in the increased secretion of resistin, and thus, GIP activates phosphoinositide 3-kinase (PI3K) and Akt/PKB (protein kinase B) through secreted resistin, thereby suppressing AMP-activated protein kinase (AMPK) in adipocytes, a key transcriptional factor in fatty acid oxidation [29,30]. Resistin is known as an adipose tissue-specific secretory factor, participating in the pathogenesis of insulin resistance, adipogenesis, and inflammation in mice [31,32]. Leptin is also a peptide hormone mainly expressed in adipose tissue, and can control the production and activation of pro-inflammatory cytokines such as TNF-α, IL-6, and IL-12 with the consequent amplification of inflammation and the development of liver fibrosis [33,34]. Previous human studies have shown that NAFLD patients have increased circulating resistin and leptin that it is correlated with insulin resistance, obesity, and the histological severity of the disease [34,35]. We also found that plasma GIP, resistin, and leptin levels, as well as the leptin/adiponectin ratio, were increased in HFD-fed mice, but SL and SLG reversed the HFD-induced increase in the plasma levels of GIP, resistin, and leptin, in addition to the leptin/adiponectin ratio (Figure 4G,H). The leptin/adiponectin ratio has been proposed as a potential surrogate biomarker for the diagnosis of metabolic diseases [36]. These observations suggest that the decrease in plasma GIP, resistin, and leptin levels is partially linked with glucose homeostasis, an increase in energy expenditure, and a decrease in the pro-inflammatory response, leading to the prevention of obesity, consequent insulin resistance, and hepatic steatosis induced by HFD.

An increase in obesity-associated inflammation can also contribute to the development of insulin resistance and hepatic steatosis [37]. It is well known that, in an obese state, the enlarged adipose tissue leads to the dysregulated secretion of adipokines and cytokines. The pro-cytokines reach metabolic tissues such as liver and muscle, and modify not only glucose and lipid metabolism, but also inflammatory responses, thereby contributing to metabolic syndrome. High levels of circulating TNF-α have been found in patients with obesity and NAFLD, and its levels are closely correlated with liver disease severity [38,39]. Moreover, circulating levels of IL-1β were demonstrated to predict T2DM when in conjunction with circulating IL-6 [40]. A previous study by Nov O [41] demonstrated that by promoting adipose inflammation and limiting fat tissue expandability, IL-1β supports ectopic fat accumulation in hepatocytes and adipose-tissue macrophages, contributing to impaired fat-liver crosstalk in nutritional obesity. In addition, IL-6 and PAI-1 are also pro-inflammatory cytokines synthesized by adipocyte, and its levels in plasma are increased in obesity and insulin resistance [42,43]. Interestingly, in the present study, SL and SLG significantly decreased plasma TNF-α, IL-1β, IL-6, and PAI-1 levels, resulting in a reduced inflammatory response, which was associated with the noticeable improvement in adiposity, insulin resistance, and hepatic steatosis by SL and SLG.

## 4. Conclusions

**Figure 5:**
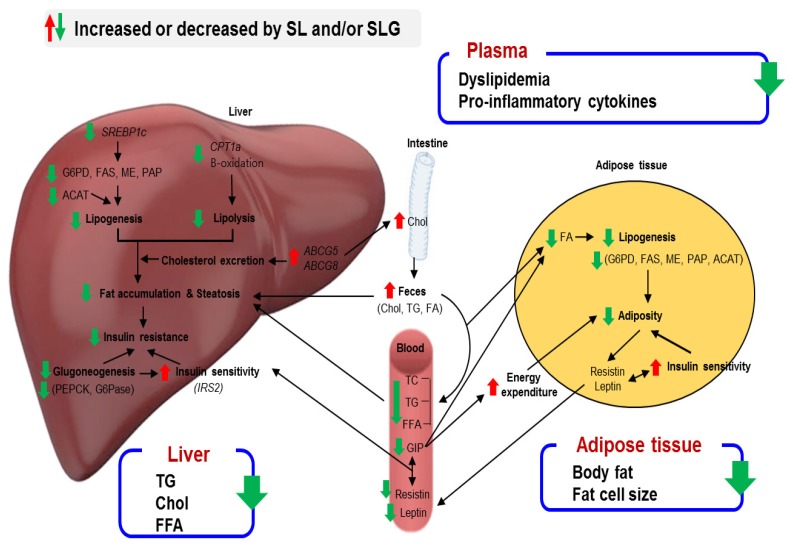
Proposed mechanism for SL and SLG regarding anti-obesity effects. SL and SLG altered the hepatic lipid and glucose metabolizing factors and decreased lipid absorption by increasing fecal lipid contents, thereby preventing hepatic steatosis via the reduction of the hepatic lipid load and eventually improving insulin resistance. In addition, SL and SLG reduced adiposity by suppressing adipocyte lipogenesis, while increasing the energy expenditure through decreasing the plasma GIP level, which is associated with a decrease in plasma pro-inflammatory cytokine levels.

The data obtained from our animal study indicate that SL and SLG can suppress DIO and modulate obesity-associated metabolic disorders such as insulin resistance and hepatic steatosis. SL and SLG prevent adiposity and dyslipidemia by suppressing the lipogenesis and the absorption of dietary fat, while increasing biliary sterol excretion and energy expenditure, which contributes to the improvement of both hepatic steatosis and lipotoxicity. SL and SLG also prevent insulin resistance by improving inflammation and decreasing gluconeogenesis. In this study, the anti-metabolic effect of SL and SLG are similarly presented, and these results thus suggest that the effect of seabuckthorn leaves may be caused by its flavonoid glycosides, including isorhamnetin-3-glucoside and quercetin-3-glucosdie. Moreover, there was no synergic effect between flavonoid glycosides and other components in seabuckthorn leaves. Figure 5 illustrates the possible mechanisms of the effects of SL and/or SLG for antiobesity. Taken together, the present findings suggest that seabuckthorn leaves, especially its flavonoid glycosides, ameliorates the deleterious effects of DIO and its metabolic complications such as adiposity, dyslipidemia, inflammation, hepatic steatosis, and insulin resistance. 

## Figures and Tables

**Table 1:** ^1^H- and ^13^C-NMR spectral data of two flavonoids isolated from seabuckthorn leaves (SL) (600 MHz).

Position	Isorhamnetin 3-Glucoside	Quercetin 3-Glucoside
^1^H-NMR		
6	6.43 (H, br s)	6.45 (H, *d*, *J* = 2.4 Hz)
8	6.72 (H, br s)	6.73 (H, *d*, *J* = 2.4 Hz)
2′	7.92 (H, *d*, *J* = 1.3 Hz)	7.71 (H, *d*, *J* = 1.8 Hz)
3′	-	-
5′	6.88 (H, *d*, *J* = 8.4 Hz)	6.85 (H, *d*, *J* = 7.8 Hz)
6′	7.60 (H, *dd*, *J* = 1.3 & 8.9 Hz)	7.60 (H, *dd*, *J* = 1.8 & 7.8 Hz)
Glu 1”	5.44 (H, *d*, *J* = 6.6 Hz)	5.20 (H, *d*, *J* = 7.2 Hz)
2”~6”	3.18 ~ 3.70	3.21 ~ 3.72
OCH_3_	3.93 (3H, s)	
^13^C-NMR		
2	157.57	158.00
3	135.32	135.72
4	177.79	179.68
5	161.71	162.85
6	100.06	100.58
7	165.12	163.63
8	94.95	95.47
9	158.63	159.60
10	105.66	105.03
1′	123.12	122.54
2′	114.38	116.13
3′	148.43	146.18
4′	150.88	150.68
5′	116.07	117.63
6′	123.81	122.54
Glu 1	103.68	103.94
2	75.94	75.74
3	78.12	77.25
4	71.50	70.08
5	78.55	75.05
6	62.59	62.57
OCH_3_	56.77	

Chemical shift in δ ppm, coupling constant (*J*) expressed in Hz in parenthesis and measured in the solvent (MeOH-d_4_). Taking TMS as an internal standard.

## Supplementary Materials

The following are available online at www.mdpi.com/2072-6643/9/6/569/s1.

## Abbreviations

ABCG	ATP-binding cassette sub-family G member
ACAT	acyl-CoA:cholesterolacyltransferase
AI	atherogenic index
Apo	apolipoprotein
BW	body weight
CPT1α	carnitine palmitoyltransferase 1α
DIO	diet-induced obese
FAS	fatty acid synthase
FFA	free fatty acid
G6Pase	glucokinase, glucose-6-phosphatase
G6PD	glucose-6-phosphate dehydrogenase
GIP	incretin hormone gastric inhibitory polypeptide
GIPR	GIP receptor
HFD	high-fat diet
HOMA-IR	homeostasis model assessment-estimated insulin resistance
IL	interleukin
IPGTT	intraperitoneal glucose tolerance test
IRS2	insulin receptor substrate 2
ME	malic enzyme
ND	normal diet
PAI-1	plasminogen activator inhibitor-1
PAP	phosphatidate phosphohydrolase
PEPCK	phosphoenolpyruvate carboxykinase
SL	seabuckthorn leaf
SLG	flavonoid glycosides from seabuckthorn leaves
SREBP1c	sterol regulatory element-binding protein 1c
T2DM	type 2 diabetes mellitus
TNF-α	tumor necrosis factor α
WAT	white adipose tissue
