# Supplementary material for: Seabuckthorn Leaves Extract and Flavonoid Glycosides Extract from Seabuckthorn Leaves Ameliorates Adiposity, Hepatic Steatosis, Insulin Resistance, and Inflammation in Diet-Induced Obesity
Source: Nutrients. 2017 Jun 2;9(6):569. doi: 10.3390/nu9060569 (PMC5490548; doi:10.3390/nu9060569)
Supplement: Supplementary file 1 [file nutrients-09-00569-s001.pdf]

Materials and Methods

Energy Expenditure

Energy expenditure was measured using an indirect calorimeter (Oxylet; Panlab, Cornella, Spain). The mice were placed into individual metabolic chambers at 25 °C, with free access to food and water. O_2 and CO_2 analyzers were calibrated with highly purified gas standards. Oxygen consumption (Vo_2) and carbon dioxide production ($V\text{co}_2$) were recorded at 3-min intervals using a computer-assisted data acquisition program (Chart 5.2; AD Instrument, Sydney, Australia) over a 24-h period, and the data were averaged for each mouse. Energy expenditure (EE) was calculated according to the following formula: EE (kcal/day/kg of body weight$^{0.75}$) = $V\text{o}_2 \times 1.44 \times (3.815 + (1.232 \times V\text{o}_2/V\text{co}_2))$.

Morphology of the Liver and Adipose Tissues

The liver and epididymal adipose tissue (eWAT) were removed from each mouse. Samples were subsequently fixed in 10% (*v*/*v*) paraformaldehyde/phosphate-buffered saline and embedded in paraffin for staining with hematoxylin and eosin. Stained areas were visualized using a microscope set at 200× magnification.

Plasma Biomarkers

Plasma lipid concentrations were determined with commercially available kits. Plasma free fatty acid (FFA) levels were measured using the Wako enzymatic kit (Wako Chemicals, Richmond, VA, USA), and triglyceride, total cholesterol, HDL-cholesterol, glutamic oxaloacetic transaminase (GOT) and glutamic pyruvic transaminase (GPT) levels were determined using Asan enzymatic kits (Asan, Seoul, Korea). Plasma apolipoprotein AI (apo AI; Eiken, Japan) and apolipoprotein B (apo B; Eiken, Japan) levels were also measured using enzymatic kits. The values of nonHDL-cholesterol, ratio of HDL-cholesterol to total cholesterol (HTR) and atherogenic index (AI) was calculated as follow: nonHDL-cholesterol = [(total-cholesterol) − (HDL-cholesterol) − (triglyceride/5)], HTR(%) = [(HDL-cholesterol/total-cholesterol) × 100, AI = [(total-cholesterol) – (HDL-cholesterol)] / (HDL-cholesterol). Plasma insulin, incretin hormone gastric inhibitory polypeptide (GIP), adipokines (resistin, leptin and

adiponectin), cytokines (tumor necrosis factor alpha (TNF-α), interleukin 1β (IL-1β), IL-6 and plasminogen activator inhibitor-1 (PAI-1)) were determined with a multiplex detection kit from Bio-Rad (Hercules, CA, USA). All samples were assayed in duplicate and analyzed with a Luminex 200 Labmap system (Luminex, Austin, TX, USA). Data analyses were done with the Bio-Plex Manager software version 4.1.1 (Bio-Rad, Richmond, CA, USA).

Fasting Blood Glucose, Intraperitoneal Glucose Tolerance Test, and Homeostatic Index of Insulin Resistance

The blood glucose concentration was measured by the glucose oxidase method using a glucose analyzer (Glucocard, Arkray, Japan) in whole blood obtained from the tail vein after food withholding for 12 h. The intraperitoneal glucose tolerance test (IPGTT) was performed at week 11. After 12 h of fasting, the mice were injected intraperitoneally with glucose (0.5 g/kg of body weight). The blood glucose level was determined from the tail vein at 0, 30, 60, and 120 min after the glucose injection. The homeostatic index of insulin resistance (HOMA-IR) was calculated according to the homeostasis assessment model as follows: HOMA-IR = (fasting glucose (mmol/L) × fasting insulin (IU/mL))/22.51.

Hepatic and Fecal Lipid Contents

Hepatic and fecal lipids were extracted as previously described [1], and then dried lipid residues were dissolved in 1 mL of ethanol for triglyceride, cholesterol, and fatty acid (FA) assays. Triton X-100 and a sodium cholate solution in distilled water were added to 200 μL of a dissolved lipid solution for emulsification. Hepatic and fecal triglyceride, cholesterol, and FA contents were analyzed with the same enzymatic kits that were used for the plasma analysis.

Preparation of Hepatic Subcellular Fractions

Hepatic and adipocyte mitochondrial, cytosolic and microsomal fractions were prepared as previously described [2]. The mitochondrial fraction was used to measure glucose-6-phosphatase (G6Pase) and β-oxidation, and the cytosolic fraction was used to measure glucose-6-phosphate dehydrogenase (G6PD), malic enzyme (ME), fatty acid synthase (FAS), glucokinase and

phosphoenolpyruvate carboxykinase (PEPCK) activities. The microsomal fraction was used to measure phosphatidate phosphohydrolase (PAP) and acyl-CoA:cholesterolacyltransferase (ACAT) activities. The protein concentrations were determined using the Bradford method.

Glucose- and Lipid-Regulating Enzyme Activity

Glucose-6-phosphate dehydrogenase (G6PD) [3], fatty acid synthase (FAS) [4], malic enzyme (ME) [5], and phosphatidate phosphohydrolase (PAP) [6] activities were measured as previously described. Glucose-6-phosphatase (G6Pase) activity was determined using the method of Alegre et al. [7]. Phosphoenolpyruvate carboxykinase (PEPCK) activity was monitored in the direction of oxaloacetate synthesis using a spectrophotometric assay developed by Bentle and Lardy [8]. Fatty acid β-oxidation was measured spectrophotometrically by monitoring the reduction of NAD to NADH in the presence of palmitoyl-CoA as described by Lazarow [4], with a slight modification.

Analysis of Gene Expression

The liver were homogenized in the TRIzol reagent (Invitrogen, Grand Island, NY, USA), and total RNA was isolated according to the manufacturer's instructions. The total RNA was converted to cDNA using the QuantiTect Reverse Transcription kit (Qiagen Gmbh, Hilden, Germany). mRNA expression was quantified by quantitative real-time polymerase chain reaction (PCR) using the QuantiTect SYBR Green PCR kit (Qiagen) and SDS7000 sequence detection system (Applied Biosystems, CA, USA). Each cDNA sample was amplified using primers for the glyceraldehyde-3-phosphate dehydrogenase (*GAPDH*) gene) labeled with SYBR green dye.

The amplification was performed as follows: 10 min at 90 °C, 15 s at 95 °C, and 60 s at 60 °C for a total of 40 cycles. The cycle threshold (Ct) was defined as the cycle at which a statistically significant increase in the SYBR green emission intensity occurred. The Ct data were normalized relative to those for the housekeeping gene, *GAPDH*, which is stably expressed in mice. Relative gene expression was calculated with the $2^{\Delta\Delta Ct}$ method [9].

Primer

The primer were designed using a Primer 5.0 software (Primer-E Ltd., Plymouth, UK), SREBP1c (Forward: 5′- GGA GCC ATG GAT TGC ACA TT-3′, Reverse: 5′-CCT GTC TCA CCC CCA GCA TA-3′), CPT1α (Forward: 5′-ATC TGG ATG GCT ATG GTC AAG GTC-3′, Reverse: 5′-GTG CTG TCA TGC GTT GGA AGT C-3′), ABCG5 (Forward: 5′- TCA ATG AGT TTT ACG GCC TGA A-3′, Reverse: 5′- GCA CAT CGG GTG ATT TAG CA-3′), ABCG8 (Forward: 5′- GCA ATG CCC TCT ACA ACT CCT T-3′, Reverse: 5′- GAG GAA CGA CAG CTT GGA GAT C-3′), IRS2 (Forward: 5′- CCC ATG TCC CGC CGT GAA G-3′, Reverse: 5′- CTC CAG TGC CAA GGT CTG AAG G-3′), and GAPDH (Forward: 5′-ACA ATG AAT ACG GCT ACA GCA ACA G-3′, Reverse: 5′-GGT GGT CCA GGG TTT CTT ACT CC-3′).

Statistical Analysis

The parameter values were expressed as the mean (standard error of the mean (SEM)). Significant differences between the ND and HFD groups were determined by student's t-test and significant differences among the HFD, SL and SLG groups were determined by one-way ANOVA using the SPSS program (SPSS Inc., Chicago, IL). Results were considered statistically significant at $p < 0.05$.

Reference

1. Folch, J., Lees, M., Sloan-Stanley, G. H., A simple method for isolation and purification of total lipids from animal tissues. *J. Biol. Chem*. 1957, 226, 497–409.
2. Davidson, A. L., Arion. W. J., Factors underlying significant underestimations of glucokinase activity in crude liver extracts: physiological implications of higher cellular activity. *Arch. Biochem. Biophys.* 1987, 253, 156–167.
3. Rudack, D.; Chisholm, E.M.; Holten, D. Rat liver glucose 6-phosphate dehydrogenase. Regulation by carbohydrate diet and insulin. *J. Biol. Chem.* **1971**, *246*, 1249–1254.
4. Lazarow, P.B. Assay of peroxisomal beta-oxidation of fatty acids. *Methods Enzymol.* **1981**, *72*, 315–319.

5. Ochoa, S. Malic enzyme: Malic enzymes from pigeon and wheat germ. In *Methods in Enzymology*; Colowick, S.P., Kaplan, N.O., Eds.; Academic Press: New York, NY, USA, 1955; Volume 1, pp. 323–326.
6. Walton, P.A.; Possmayer, F. Mg2-dependent phosphatidate phosphohydrolase of rat lung: Development of an assay employing a defined chemical substrate which reflects the phosphohydrolase activity measured using membrane-bound substrate. *Anal. Biochem.* **1985**, *151*, 479–486.
7. Alegre, M.; Ciudad, C.J.; Fillat, C.; Guinovart, J.J. Determination of glucose-6-phosphatase activity using the glucose dehydrogenase-coupled reaction. *Anal. Biochem*. **1988**, *173*, 185–189.
8. Bentle, L.A.; Lardy, H.A. Interaction of anions and divalent metal ions with phosphoenolpyruvate carboxykinase. *J. Biol. Chem.* **1976**, *251*, 2916–2921.
9. Schmittgen, T.D.; Livak, K.J. Analyzing real-time PCR data by the comparative C(T) method. *Nat. Protoc*. **2008**, *3*, 1101–1108.
